# The influence of sex-linked genetic mechanisms on attention and impulsivity

**DOI:** 10.1016/j.biopsycho.2011.09.011

**Published:** 2012-01

**Authors:** Simon Trent, William Davies

**Affiliations:** Behavioural Genetics Group, MRC Centre for Neuropsychiatric Genetics and Genomics, Neuroscience and Mental Health Research Institute, Schools of Psychology and Medicine, Cardiff University, United Kingdom

**Keywords:** Sexual dimorphism, Neuropsychiatric disorders, Attention, Impulsivity, Attention deficit hyperactivity disorder, Sex chromosomes, Autism, SRY, MAOA, Steroid sulfatase, Neurosteroids, Turner syndrome, COMT

## Abstract

It is now generally agreed that there are inherent sex differences in healthy individuals across a number of neurobiological domains (including brain structure, neurochemistry, and cognition). Moreover, there is a burgeoning body of evidence highlighting sex differences within neuropsychiatric populations (in terms of the rates of incidence, clinical features/progression, neurobiology and pathology). Here, we consider the extent to which attention and impulsivity are sexually dimorphic in healthy populations and the extent to which sex might modulate the expression of disorders characterised by abnormalities in attention and/or impulsivity such as attention deficit hyperactivity disorder (ADHD), autism and addiction. We then discuss general genetic mechanisms that might underlie sex differences in attention and impulsivity before focussing on specific positional and functional candidate sex-linked genes that are likely to influence these cognitive processes. Identifying novel sex-modulated molecular targets should ultimately enable us to develop more effective therapies in disorders associated with attentional/impulsive dysfunction.

## Highlights

► Attention and impulsivity are sexually dimorphic in healthy populations. ► Review how sex modulates the expression of disorders like ADHD and autism. ► Genetic mechanisms that underlie sex differences in attention/impulsivity discussed. ► Focus on candidate sex-linked genes likely to influence these cognitive processes.

## 1 Introduction

Ever more sophisticated neuroscientific approaches have strengthened the notion of a considerable degree of neural sexual dimorphism amongst healthy individuals (Cahill, 2006; Jazin and Cahill, 2010). Sex differences in structure/function have been observed for a number of brain regions underlying higher cognitive and ‘emotional’ processes including the hippocampus (Goldstein et al., 2001; Madeira and Lieberman, 1995), the amygdala (Hamann, 2005; Mechelli et al., 2005) and the prefrontal cortex (PFC) (Goldman et al., 1974; Shansky et al., 2004). Moreover, the sexes seem to differ significantly with respect to their neurochemical systems (Craft, 2003; Galanopoulou, 2005), notably the monoamine system (Nishizawa et al., 1997; Robinson et al., 1977).

There is perhaps even more compelling evidence for sexual dimorphic features in many neuropsychiatric disorders, notably in terms of their incidence, their clinical features and progression, and in their underlying pathology (Holden, 2005; Klein and Corwin, 2002). A large proportion of neuropsychiatric disorders, including attention deficit hyperactivity disorder (ADHD), autism, pathological gambling, depression and schizophrenia, are characterised by impairments in higher cognitive functions; two domains which are consistently reported as being abnormal in such disorders are attention and impulsivity (American Psychiatric Association, 1994; Blaszczynski et al., 1997; Chen and Faraone, 2000; Moritz et al., 2002; Simonoff et al., 2008). Moreover, there is substantial evidence to suggest that attention/impulsivity may be influenced by common brain substrates such as regions of prefrontal cortex (Bush, 2010; Cardinal, 2006; Coull et al., 1996). Here, we examine the evidence for these two neuropsychological domains being sexually dimorphic in both healthy individuals and individuals with a psychiatric diagnosis using PubMed searches (keywords included ‘sex’ or ‘gender differences’, ‘attention’ or ‘attentional’, ‘impulsive’ or ‘impulsivity’, ‘ADHD’, ‘stop signal’, ‘delay discounting’ or ‘aversion’, ‘response inhibition’ and others). Furthermore, we suggest genetic mechanisms that may mediate any sex bias and three candidate sex-linked genes are considered. Much of our discussion will focus on ADHD, the archetypal disorder of attention and impulsivity (American Psychiatric Association, 1994). It is estimated that greater than 75% of ADHD diagnoses are made in males (Pastor and Reuben, 2008; Schneider and Eisenberg, 2006), and whilst this figure may be partly confounded by ascertainment and referral biases (Biederman et al., 2002; Derks et al., 2007), it is also likely to reflect some sex-specific difference in biological vulnerability (Waddell and McCarthy, 2011).

## 2 A brief introduction to attention and impulsivity

All animals live in a world of competing, multiple and simultaneous stimuli that must be resolved in order so that they behave adaptively. Attention represents the ability to select from a plethora of stimuli, responses, memories and thoughts, and in doing so, disregard any that are behaviourally irrelevant (Raz, 2004); ‘attention’ may be regarded as a unitary description of three neurobiologically disparate attentional control systems: ‘alerting’, ‘orienting’ and ‘executive’ (Posner and Petersen, 1990; Raz and Buhle, 2006). ‘Alerting’ relates to preparedness for an impending stimulus by achieving and maintaining an alert state (it may also be termed as ‘sustained attention’ or ‘vigilance’). Parallel neuroimaging and neuropsychological studies have revealed that the alert state corresponds to activity in the prefrontal and parietal cortices, particularly in the right hemisphere (Coull et al., 1996), and is dependent upon the noradrenaline system (Beane and Marrocco, 2004; Marrocco et al., 1994). Orienting (also known as ‘scanning’ or ‘selection’) refers to the ability to select information from multiple sensory stimuli, and has been reported to be associated with superior parietal cortex activity (Corbetta et al., 2000), and superior colliculus activity (overt attentional shifts; Corbetta, 1998). The disengagement of attention when a target occurs at an uncued location is thought to involve the temporo-parietal junction and superior temporal lobe (Friedrich et al., 1998; Karnath et al., 2001). The cholinergic system is thought to play an important role in orienting through its effects on the parietal cortex (Beane and Marrocco, 2004; Parasuraman et al., 1992; Tales et al., 2002; Voytko et al., 1994) and cholinergic agonists have been shown to reduce neural activity and reaction times on invalidly cued trials (Thiel et al., 2005). ‘Executive attention’ (also referred to as ‘supervisory’, ‘selective’ and ‘focussed attention’) is the complex monitoring and resolution of conflict between diverse brain regions and is classically measured through tasks such as the Stroop task (Stroop, 1935) which possess an incompatibility between the dimensions of the stimulus that must be resolved. In the Stroop test, the incongruent condition of the word red printed in blue ink must be resolved such that subjects correctly report the colour of the ink and inhibit the prepotent process of stating the printed word. Neuroimaging studies have identified the anterior cingulate cortex (ACC) as a key brain region in executive attention given that the dorsal ACC is consistently activated in ‘conflict’ tasks (Fan et al., 2003a). It is currently unclear whether the ACC monitors conflict by engaging the dorsolateral prefrontal cortex (Botvinick et al., 2004), whether it resolves conflict (at response, not stimulus level) (Liu et al., 2004; Milham et al., 2001) or whether it does both (Bush et al., 2000). The ACC and dorsolateral prefrontal cortex are both targets of the mesocortical dopamine pathway (Marrocco and Davidson, 1998), so it has been postulated that the executive attentional network is especially sensitive to dopaminergic function and pharmacological manipulation (Raz, 2004; Raz and Buhle, 2006).

As with attention, a single definition that encapsulates all aspects of impulsivity has so far proved elusive (Winstanley et al., 2006) and this likely derives from its considerable heterogeneity (Evenden, 1999b). Broadly, impulsivity describes several phenomena pertaining to ‘action without forethought’ that forms part of everyday behaviour and, in some instances, contributes to adverse states such as drug addiction (Moeller et al., 2001), gambling (Steel and Blaszczynski, 1998) and ADHD (Sagvolden et al., 1998). Impulsivity may be sub-classified into at least two distinct theoretical entities: ‘impulsive action’ (associated with a lack of behavioural inhibition) and ‘impulsive choice’ (decision-making without appropriate deliberation of the alternative options) (Evenden, 1999a). Impulsive action includes premature/mistimed actions and actions that are difficult to control/suppress and are commonly measured experimentally in humans and rodents using Go/No-go and Stop-signal Reaction Time (SSRT) tasks (Band and van Boxtel, 1999; Eagle and Robbins, 2003; Harrison et al., 1999). Impulsive choice is exemplified by aversion to delayed reward (Dalley et al., 2008) and is often measured experimentally using delay-discounting paradigms whereby impulsivity is observed as a greater tendency towards a small, immediate reward over a larger, delayed reward (Cardinal et al., 2004). However, the theoretical classification of impulsivity often overlooks the complicated and myriad forms of manifestation at the phenotypic level, such as the concomitant expression of impulsivity and aggression, known as impulsive aggression, which is commonly observed in antisocial personality and borderline personality disorders (Seo et al., 2008).

Numerous studies investigating the neuroanatomical circuitry of impulsivity have demonstrated the key role that the frontal cortex plays in humans (Brass and von Cramon, 2002; Cardinal, 2006; Dove et al., 2000; Mecklinger et al., 1999). This is particularly evident in human subjects with damage to the right inferior frontal gyrus and ventromedial frontal cortex, who subsequently display deficits in stop-signal inhibition (Aron et al., 2003) and poor decision-making in a gambling task (Bechara et al., 1994), respectively. Systematic lesion studies in rats have begun to dissociate between regions of the brain underlying different aspects of impulsivity (reviewed in Winstanley et al., 2006). To date, these implicate the infralimbic, anterior cingulate and orbitofrontal regions of the frontal cortex (but not the prelimbic) and the medial striatum as mediators of impulsive action as measured by the 5-choice serial reaction time task (5-CSRTT), where rats must refrain from responding to a stimulus prematurely. These lesion studies have provided convincing evidence that the neurobiology underlying impulsive action and impulsive choice may be dissociable to some extent (Winstanley et al., 2006), in that whilst lesions of the nucleus accumbens increase both subtypes of impulsivity (Cardinal et al., 2001; Christakou et al., 2004), lesions to either the orbitofrontal cortex or subthalamic nucleus increase impulsive actions, but result in an increased tolerance to delay (Winstanley et al., 2004, 2005).

Research into the underlying neurochemistry of impulsivity has focussed on dopaminergic, serotonergic, noradrenergic and glutamatergic pathways (Cardinal et al., 2004; Eagle and Baunez, 2010; Pattij and Vanderschuren, 2008). The dopaminergic system has been implicated in impulsivity phenotypes by the observation that psychostimulant drugs which enhance dopamine release from dopaminergic terminals and/or prevent dopamine reuptake (e.g. methylphenidate and d-amphetamine) can be used to treat disorders of impulsivity such as ADHD (Sandoval et al., 2002; Tripp and Wickens, 2009; Volkow et al., 1998). d-Amphetamine enhances stop-signal task performance in both humans and rats (de Wit et al., 2000; Feola et al., 2000), although it has been shown to increase premature responding in the 5-CSRTT and other similar tasks (Cole and Robbins, 1987; Pattij and Vanderschuren, 2008; van Gaalen et al., 2009; Wiskerke et al., 2011). Meanwhile, d-amphetamine has been shown to decrease impulsive choice in delay-discounting tasks (de Wit et al., 2002; Isles et al., 2003), but conversely, an increase in impulsive choice has also been demonstrated (Wiskerke et al., 2011). There is generally considered to be an inverse correlation between 5-HT levels and impulsive action (Crean et al., 2002; Harrison et al., 1997; Walderhaug et al., 2002), although it is unclear if the same is true of delay-aversion (Cardinal, 2006). The involvement of noradrenergic and glutamatergic pathways in impulsivity has been suggested by the ability of noradrenaline reuptake inhibitors (e.g. atomoxetine) to reduce impulsive action across species (Chamberlain et al., 2006; Robinson et al., 2008) and NMDA antagonists/metabotropic glutamate receptor antagonists to modulate impulsive action (Higgins et al., 2003; Sukhotina et al., 2008), respectively.

## 3 Sex differences in attention and impulsivity in healthy individuals

*A priori*, one might anticipate sex differences in attention and impulsivity, given that brain regions underpinning these functions (described above) differ considerably between the sexes in terms of their development and ongoing function (Bland et al., 2005; Duff and Hampson, 2001; Goldman et al., 1974; Shansky et al., 2004). The scientific literature documents a surprisingly small number of objective neuropsychological studies assaying sex differences in attention and impulsivity in healthy individuals. Several studies have suggested a female advantage with respect to executive attention/response inhibition as indexed by Stroop task performance (Moering et al., 2004; van Boxtel et al., 2001; Van der Elst et al., 2006), possibly related to sex differences in corpus callosum morphology throughout adolescence (Silveri et al., 2006); however, other studies have not recognised an equivalent behavioural effect (Klein et al., 1997; Swerdlow et al., 1995). In contrast, males may outperform females on an oddball task tapping visuospatial selective attention, and may activate different neural structures: during this task, men showed increased activation in the left hemispheric inferior temporo-parietal region whilst women exhibited increased activation of the right-hemispheric inferior frontal, insula, putamen and superior temporal regions (Rubia et al., 2010).

With regard to impulsive action, behavioural performance on the SSRT task does not seem to be modulated to any great extent by sex; however, sex does seem to influence the function of several brain regions associated with behavioural output including the cingulate cortex, corpus callosum, the globus pallidus and the thalamus, suggesting either diverse neural strategies or compensatory brain mechanisms which may act to ensure sex-matched performance (Huster et al., 2011; Li et al., 2006). No similar studies have been conducted to assess sexual dimorphism in impulsive choice directly. However, impaired planning and therefore decision-making are clearly implicit in impulsivity and studies have shown sexual dimorphism in terms of the underlying neural substrates of decision-making (Bolla et al., 2004; Tranel et al., 2005). Here, males have been shown to outperform women in the extensively used Iowa Gambling Task (Overman, 2004; Reavis and Overman, 2001). Using this task, right hemisphere PFC lesions impaired decision-making in men, but not in women, whereas the reverse was true of left hemisphere lesions (Tranel et al., 2005).

## 4 Sex differences in attention and impulsivity in neuropsychiatric disorders

### 4.1 Attention deficit hyperactivity disorder (ADHD)

ADHD is a common neurodevelopmental disorder (Polanczyk et al., 2007) with a strong genetic basis (Wallis et al., 2008), characterised by deficits in attention, pathological impulsivity and extreme hyperactivity. Individuals with ADHD are typically diagnosed as having one of the three subtypes: the inattentive subtype (showing inattentive symptoms in isolation and often associated with an introspective, distractible, disorganised persona), the hyperactive-impulsive subtype (showing hyperactivity and impulsive symptoms, but few, if any, inattentive symptoms, often associated with an energetic, extrovert, thrill-seeking, impatient, and potentially aggressive persona) and combined subtype (an amalgam of the previous two subtypes) (American Psychiatric Association, 1994). The phenotypic dissociation between the inattentive subtype, and the remaining subtypes has led some researchers to propose that they should be regarded as nosologically separate entities, underpinned by discrete neurobiologies, and sensitive to distinct treatment regimes (Larsson et al., 2006; Polanczyk et al., 2007; Solanto et al., 2009; Stein et al., 2003). The sex discrepancy in overall ADHD prevalence is considerable (substantially increased prevalence in males) (Holden, 2005; Swanson et al., 1998), and furthermore, appears to be subtype-specific: inattentive ADHD is most prevalent amongst girls (Biederman et al., 2002), whereas hyperactive-impulsive and combined subtypes are thought to be more common in boys (Adler et al., 2008). Thus, sex differences in presentation of the disorder may feasibly account for sex-specific differences in referrals and diagnosis (Biederman et al., 2002; Lahey et al., 1994).

In terms of symptomatology, females diagnosed with ADHD may present with lower ratings of hyperactivity, inattention, impulsivity and externalising problems (e.g. aggression) than ADHD males, but greater intellectual impairments and more internalising problems (e.g. affective, eating and somatisation disorders) (Gershon, 2002). In contrast, abuse and criminality appear to be more prevalent amongst ADHD males (Gershon, 2002). To date, there has been little research explicitly examining whether male and female ADHD patients differ in their performance on attention and impulsivity tasks. One study, employing both the Stroop and the stop-signal tasks, found no significant sex difference within the ADHD patient population (Rucklidge and Tannock, 2002). In contrast, a recent meta-analysis of stop-signal studies found a borderline effect of gender, whereby behavioural inhibition in male ADHD patients (compared with male healthy controls) was more severely impaired than in female ADHD patients (compared with female healthy controls) (Lipszyc and Schachar, 2010).

Consistent evidence for sex differences in brain structure/function within ADHD cohorts has been limited (Castellanos et al., 2001; Hill et al., 2003), in agreement with the idea of relatively subtle differences in symptomatology and neuropsychological function between ADHD males and females. However, it is also possible that true sex differences in these domains may be obscured by confounds including the age of the patients being scanned, the small study sizes, their different treatment regimes and of the variability inherent in diagnosis of the disorder. Indeed, a recent study by Valera et al. (2010) in which some of these confounds were addressed has revealed that whilst adult ADHD males and females did not differ in their behavioural performance on a working memory task, male ADHD cohorts exhibited reduced activity in a number of pertinent brain structures (frontal, temporal and cerebellar regions) relative to control males; in contrast, females with ADHD did not show a similar attenuation in activity relative to female controls. First-line treatments for ADHD, such as the psychostimulant methylphenidate, are thought to exert their primary effects through enhancing dopaminergic neurotransmission (thereby counteracting dysregulated dopamine pathways in ADHD) (Heal et al., 2009) and, although they may be metabolised differently in males and females, appear to be equally efficacious in both sexes (Cornforth et al., 2010; Gunther et al., 2010; Rucklidge, 2010). However, a recent study has uncovered between-sex differences in the efficacy of a newer ADHD treatment, atomoxetine (which acts primarily as a noradrenaline re-uptake inhibitor), with better outcomes in females compared with males (Marchant et al., 2011).

### 4.2 Other neuropsychiatric disorders

Autism spectrum disorders (ASDs) are characterised by abnormalities in social function and communication, excessive anxiety and high levels of repetitiveness (American Psychiatric Association, 1994; Lord et al., 2001). As with ADHD, family, twin and adoption studies have indicated that vulnerability to ASDs is influenced to a large extent by genetic factors (Abrahams and Geschwind, 2008; Bailey et al., 1995; Folstein and Rutter, 1977). Although ASDs are not generally considered disorders of inattention or impulsivity *per se*, it is estimated that 30–80% of ASD children meet symptom criteria for ADHD (Carpenter Rich et al., 2009; Hattori et al., 2006; Lee and Ousley, 2006; Simonoff et al., 2008). ASDs disproportionately affect males (male:female prevalence ratio is ∼4:1) (Fombonne, 2002) and it has been proposed that autistic behaviours and neurobiology represent extremes of those which distinguish males from females (the ‘Extreme Male Brain Theory’; Baron-Cohen, 2002; Baron-Cohen et al., 2005). Whilst studies explicitly comparing attention and impulse control in male and female ASD subjects are rare, there is some evidence that female patients may exhibit more inattentive symptoms (Holtmann et al., 2007) and poorer response inhibition as indexed by SSRT task performance (Lemon et al., 2011) than their male counterparts. The neurobiological substrates underlying these behavioural differences in this clinical group remain to be elucidated; candidate brain regions may include the temporal lobe and cerebellar grey matter (both reduced in size in female ASD subjects) (Bloss and Courchesne, 2007).

Addictive behaviours, such as gambling and drug abuse, can be conceptualised as impulsive choices, whereby smaller, immediate rewards are favoured over larger, delayed rewards (Petry and Casarella, 1999). Pathological gambling, the maladaptive behaviour of gambling in spite of adverse consequences (Alessi and Petry, 2003), is characterised by high levels of impulsivity (American Psychiatric Association, 1994; Blaszczynski et al., 1997; Petry, 2001; Steel and Blaszczynski, 1998) and frequently co-occurs with ADHD (Derevensky et al., 2007; Specker et al., 1995). Furthermore, gender differences in gambling have been described in aspects of incidence (males are at an increased risk), age of onset (earlier age of onset in males), course (females have quicker progression to pathological gambling), gambling preferences and comorbidity with alcohol abuse (more common in men) (Johansson et al., 2009; Martins et al., 2008; Tavares et al., 2001). Despite a growing trend towards research into female gamblers (Martins et al., 2002), we are unaware of any study specifically examining sex differences in impulsivity levels amongst pathological gamblers. Drug abuse is the acquisition and initiation of substances such as opiates, nicotine, alcohol, cocaine and psychomotor stimulants, all of which may lead to addiction (Le Moal and Koob, 2007). Similar to pathological gambling, drug abuse is closely associated with impulsive behaviour (Perry and Carroll, 2008), such that drug abusers are significantly more impulsive than controls (Kirby et al., 1999; Madden et al., 1997; Vuchinich and Simpson, 1998). In fact, the association between impulsivity and drug abuse may explain the high comorbidity between drug abuse and ADHD (Schubiner, 2005), especially given that the inattentive ADHD subtype was found to be less of a risk for substance abuse than the hyperactive-impulsive subtype (Elkins et al., 2007). Sex differences have been reported in all facets of human and animal drug abuse including initiation, escalation, addiction and relapse following withdrawal (Becker and Hu, 2008; Carroll et al., 2004; Lynch et al., 2002; Roth et al., 2004). For example, the rate of escalation of drug use and risk of relapse following abstinence is greater in females compared to males (Brady and Randall, 1999; Breese et al., 2005; Carpenter et al., 2006; Hernandez-Avila et al., 2004; Mann et al., 2005). Meanwhile, sex differences in impulsivity have been reported in human drug abusers (Lejuez et al., 2007) and rat models of drug abuse (Anker et al., 2008; Perry et al., 2007), with greater impulsivity found amongst drug abusing females compared with corresponding drug abusing males.

## 5 Genetic mechanisms of sexual differentiation of the brain

Ultimately, sex differences in behaviour and cognition must stem from the fact that the two sexes inherit different chromosomal complements: males inherit one cognition-gene rich X chromosome (invariably from their mother) (Zechner et al., 2001), and a Y chromosome from their father, whereas females inherit two X chromosomes, one from either parent. Accumulating data from the elegant mouse models has shown that in mammals, genes on the sex chromosomes (the X and Y) may influence neurobiology directly (through influencing neurodevelopment and/or ongoing brain function), or may influence neurobiology indirectly through affecting some intermediary pathway (notably gonadal hormone secretion and function) (Arnold and Chen, 2009; Dewing et al., 2003, 2006). There is evidence for multiple gene expression differences between regions of male and female brains relevant to attention and impulsivity i.e. the dorsolateral prefrontal cortex, the anterior cingulate cortex and the cerebellum (Vawter et al., 2004); it is likely that these expression differences are the upshot of a complex interaction between sex chromosome complement and hormonal milieu.

Multiple lines of evidence have suggested a strong genetic component to attention and impulsivity phenotypes, including data from family, twin and adoption studies for disorders of attention/impulsivity such as ADHD (Faraone et al., 2005; Sharp et al., 2009; Thapar et al., 2007), and the fact that rats can be selectively bred so as to exhibit inattentive and impulsive phenotypes (Moreno et al., 2010; Sagvolden et al., 2005). In the case of twin studies, both qualitative and quantitative genetic sex differences (measured as differences in correlations between opposite sex dizygotic twins with same-sex dizygotic twins and between male monozygotic twins with female monozygotic twins, respectively) have been reported in the hyperactivity levels of 7-year olds (Saudino et al., 2005). However, this was only evident in same-teacher scores and not parent scores, whilst two other twin studies that specifically assessed ADHD symptoms, demonstrated a lack of quantitative and qualitative genetic sex differences using the Revised Connor's Parent Rating Scale (Greven et al., 2011; Kuntsi et al., 2005) and a meta-analysis of twin studies did not reveal an effect of sex on ADHD symptom dimensions (Nikolas and Burt, 2010).

Evidence for the involvement of sex-linked genes specifically in mediating altered attention/impulsivity phenotypes has come from a combination of comparing males and females on measures of attention/impulsivity (described above), studying cohorts with unusual sex chromosome constitutions, from studying individuals with discrete mutations on the X or Y chromosomes, from linkage/association studies and from work in animal models. For example, using an elegant animal model, the so-called ‘four-core genotypes’ cross in which the direct effects of sex-linked genes on neurobiology may be dissociated from their downstream effects on gonadal hormone-mediated effects on neurobiology (De Vries et al., 2002), it has been shown that chromosomally female mice (XX) showed faster food-reinforced instrumental habit formation than chromosomally male mice (XY), irrespective of their gonadal type (Quinn et al., 2007). This finding suggests that the greater escalation of drug use in females, and more rapid progress to addiction in this sex, may be dependent upon the actions of sex-linked genes.

There are three general mechanisms through which genes on the sex chromosomes may influence sex-specific neurobiology (Davies et al., 2005). First, genes on the Y chromosome can only be expressed in male brain cells (Kopsida et al., 2009). Second, X-linked genes that escape the process of X-inactivation (Ohno et al., 1959; Reik and Lewis, 2005) (∼20% of all X-linked genes in man) will be expressed more highly in female than male brain as a consequence of the fact that females possess two copies of any given X-linked gene, whereas males only possess one; moreover, male hemizygosity for X-linked genes (i.e. the fact that males only possess one allele of each such gene), means that these alleles will be able to directly influence phenotype. In females, the effect of any particular X-linked allele on phenotype may be masked by the effect of the allele on the opposite X chromosome. Finally, so-called ‘imprinted’ genes on the X chromosome may, in theory, be differentially expressed in male and female brain (Davies and Wilkinson, 2006): genes expressed solely from the paternally inherited X chromosome can only be expressed in female brain (as only females inherit an X chromosome from their father), whereas genes expressed from the maternally inherited X chromosome may be expressed in both sexes, but may be more highly expressed in male brain if they are subject to X-inactivation. Whilst there are many examples of Y-linked genes and X-linked dosage-sensitive genes that influence physiology in man, currently the effect of X-linked imprinted genes on brain and behaviour in man remains theoretical given that no such genes have yet been identified. In addition to these mechanisms, it is possible that regulatory elements on the sex chromosomes may influence autosomal gene expression and that chromosomal interactions within the nucleus involving the X and Y chromosomes may influence sex-specific gene expression (Eskiw et al., 2010; Heard and Bickmore, 2007).

Perturbations to these genetic mechanisms of sexual differentiation of the brain (via chromosomal abnormalities for example) may result in abnormal attentional and/or impulsivity phenotypes. Subjects with such chromosomal abnormalities are relatively rare and their phenotype varies considerably, and as such, data from such studies should be treated with a degree of caution. Males with multiple Y chromosomes, and therefore over-dosage of Y-linked genes (most commonly karyotype 47,XYY), have been reported to exhibit relatively high rates of attentional deficits (distractibility) and ADHD (Linden and Bender, 2002; Ross et al., 2009; Ruud et al., 2005). These findings are somewhat consistent with evidence for impaired response inhibition in 47,XYY males (Ross et al., 2009). A report of a male with a major *de novo* mutation of the Y chromosome (deletion of the long arm, together with duplication of the short arm) and ADHD suggests the possibility that over-dosage of Y-linked genes on the short arm and/or absence of Y-linked genes on the long arm could be important in mediating ADHD vulnerability (Mulligan et al., 2008).

Evidence for X-linked gene dosage being important in mediating attentional/impulsive function has come from studying subjects with Turner syndrome (TS; the majority of whom possess a single X chromosome only, karyotype 45,X) and Klinefelter syndrome (KS; males possessing an additional X chromosome of either paternal or maternal origin, karyotype 47,XXY). Both of these disorders present with endocrinological abnormalities (Gravholt, 2004; Lanfranco et al., 2004), so any behavioural deficits associated with these conditions could be a direct consequence of altered gene dosage within the brain, or to brain effects mediated indirectly by systemic gonadal hormone levels. Rates of ADHD, and particularly the hyperactive-impulsive subtype, are significantly higher in TS individuals than in control 46,XX subjects (Russell et al., 2006), presumably as a consequence of haploinsufficiency (reduced dosage) for one or more X-linked genes that typically escape X-inactivation; TS subjects also show impairments across a number of neuropsychological tests taxing attention and/or impulsivity (Nijhuis-van der Sanden et al., 2003; Ross et al., 2002; Rovet and Ireland, 1994). Interestingly, 39,XO mice, a putative model for aspects of TS neurobiology (Lynn and Davies, 2007), show deficits in visuospatial attention which recapitulate those seen in TS subjects (Davies et al., 2007) implicating the few X-linked genes that escape X-inactivation in both mouse and man as candidates underlying this behavioural abnormality. The evidence for attentional/impulsivity impairments in KS subjects is less strong than that for TS; however, again this group may be at a slightly elevated risk of developing ADHD, be more distractible, and show deficits in some forms of executive function (Leggett et al., 2010; Linden and Bender, 2002; Ross et al., 2009). The combined TS and KS data suggest the possibility that altered X-linked gene dosage in either direction (either under, or over-dosage) may result in phenotypically similar outcomes.

## 6 Candidate sex-linked genes influencing attention and impulsivity

**Fig. 1:**
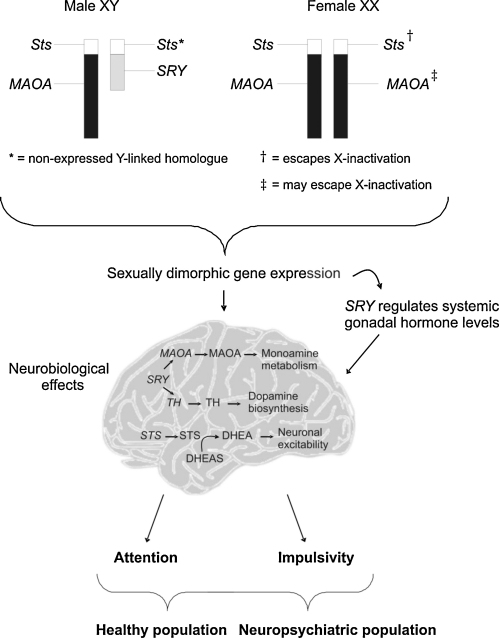
The genetic mechanisms that underlie attention and impulsivity in both the healthy and the neuropsychiatric population. The expression of sex linked genes including *SRY*, *STS* and *MAOA* are sexually dimorphic (*SRY* is only expressed in males and *STS* has a higher expression in females). As a consequence, sex differences may occur in their neural expression or indirect downstream effects on systemic gonadal hormone levels (via *SRY*). In turn, sexually dimorphic neurobiological alterations in cognitive-associated brain regions and neurotransmitter pathways such as dopamine, may result in sex specific nuances in attention and impulsive behaviour amongst the healthy population, but also the differences within the neuropsychiatric population.

The sex-linked genes *SRY*, *STS* and *MAOA* represent clear positional and/or functional candidates for effects on attentional and impulsive behaviours. For instance, *SRY*, either directly via neural expression or indirectly through downstream effects on gonadal hormone levels, may mediate dopaminergic effects linked with attention and impulsivity (Boulougouris and Tsaltas, 2008; Dewing et al., 2006). *MAOA* may similarly influence general monoaminergic function (Shih et al., 1999), whereas *STS* has been implicated in both neuropsychological domains (Kent et al., 2008; Stergiakouli et al., 2011). Below we discuss the three candidate sex-linked genes in further detail (also shown in Fig. 1).

### 6.1 SRY

One obvious candidate sex-linked gene is the sex-determining gene itself, *SRY* (Sex-determining Region on the Y) (Sekido and Lovell-Badge, 2009). *SRY* gene is a Y-linked gene (Yp11.3) (and hence male-specific) which encodes a protein with a DNA-binding motif. This protein acts as a transcription factor in the bipotential gonad of the developing fetus to induce gene expression changes which facilitate differentiation into testicular tissues (Berta et al., 1990; Kashimada and Koopman, 2010; Sinclair et al., 1990). Once formed, the Leydig cells of the testis secrete testosterone in the presence of luteinising hormone; this testosterone (and its metabolites) may then masculinise the brain through acting at androgen or oestrogen receptors (Zuloaga et al., 2008). Besides acting as a key molecular switch in the gonads, recent data has shown that SRY may also act as a transcriptional regulator in the brain. In rodents, the gene is highly expressed in the substantia nigra (SN) and ventral tegmental area (VTA) brain regions (Dewing et al., 2006; Lahr et al., 1995); these areas are highly enriched for dopaminergic neurons, which project to the frontal cortex and striatum (Thierry et al., 2000). In man, *SRY* has been reported as being expressed in adult frontal and temporal cortex, and in the medial rostral hypothalamus (Mayer et al., 1998); it is likely that, as in rodents, *SRY* is also expressed in the SN/VTA of human males, but this has yet to be investigated. The pattern of *SRY* expression prompted researchers to investigate whether its associated protein could act as a transcription factor for important genes in monoamine metabolism. Promoter-binding and immunoprecipitation assays have since demonstrated that SRY may act as a transcriptional activator for *TH* (the gene encoding the rate-limiting enzyme in dopamine biosynthesis tyrosine hydroxylase) (Milsted et al., 2004) and for *MAOA* (the X-linked gene encoding the enzyme monoamine oxidase involved in monoamine breakdown) (Wu et al., 2009); the fact that knockdown of *SRY* expression in the rat SN results in reduced TH protein provides evidence that these findings may have relevance *in vivo* (Dewing et al., 2006).

Hence, SRY represents an excellent candidate for sex-specific effects on cognitive domains that are known to be highly sensitive to dopaminergic function in the frontal cortex and striatum, including attention/impulsivity (Boulougouris and Tsaltas, 2008; Nieoullon, 2002; Pattij and Vanderschuren, 2008; Robbins and Roberts, 2007; Winstanley et al., 2006), in the normal and pathological ranges. Both ADHD and addictive behaviours are highly male-biased in their prevalence, and are associated with dopaminergic dysregulation (Andersen and Teicher, 2000; Hyman et al., 2006; Quinn et al., 2007). *SRY* could potentially also act as an indirect mediator of attention/impulsivity via its downstream effects on testosterone secretion. Testosterone levels may influence attention/impulsivity in individuals with attentional/impulsive dysfunction (Baron-Cohen et al., 2005; de Bruin et al., 2006; Martel et al., 2009), although it is less clear whether this is mirrored in healthy individuals (Bjork et al., 2001; Cherrier et al., 2002; Wolf and Kirschbaum, 2002). *SRY* overexpression represents a plausible candidate genetic mechanism underlying the increased risk of attentional problems in subjects with duplication of the short arm of the Y chromosome (Mulligan et al., 2008) and 47,XYY individuals. If *SRY* does mediate attention/impulsivity phenotypes, we might further speculate that females with Swyer syndrome who possess a 46,XY chromosomal constitution but who commonly lack a functional *SRY* gene, might display abnormalities in these domains.

Just as Y-linked genes such as *SRY* may influence sex-specific cognitive attributes, so too might X-linked genes, whether that be through the effects of increased dosage of genes escaping X-inactivation in females, or through expression of particular alleles in hemizygous males but not in homo- or heterozygous females.

### 6.2 STS

The most parsimonious explanation for the attentional impairments in individuals with TS, and in 39,XO mice, is haploinsufficiency for one or more X-linked genes that typically escape X-inactivation in both species. The fact that the 39,XO mouse deficit could be rescued in 40,XY*^X^ mice (essentially 39,XO mice with a few additional X-linked genes on the small Y*^X^ chromosome) suggested that one of the genes on this chromosome could influence attention. The most promising candidate gene on the basis of its function, and the fact that it was already known to escape X-inactivation, was *STS*, encoding the enzyme steroid sulfatase (Davies et al., 2007). Steroid sulfatase catalyses the desulfation of the various neurosteroids thus modulating their activity, e.g. dehydroepiandrosterone sulfate (DHEAS) to DHEA (Reed et al., 2005). Neurosteroids are synthesised in the brain, as opposed to the adrenal glands and gonads (Dubrovsky, 2005), and have numerous neural functions including effects on neuronal excitability (DHEAS is a potent negative allosteric modulator of GABA receptors) and gene transcription via nuclear steroid receptors (Belelli and Lambert, 2005; Rupprecht and Holsboer, 1999). In man, the *STS* gene is X-linked (Xp22.3), escapes X-inactivation (Shapiro et al., 1979) and has a non-expressed Y-linked homologue (Yen et al., 1988). *A priori*, one may expect expression to be higher in female than male tissues. Whilst there is indeed some evidence for greater activity in accessible tissues in human females (Cuevas-Covarrubias et al., 1993), and in female brain tissue from monkeys (Kriz et al., 2005) as yet there is no robust data on the expression/activity of the enzyme in regions of male and female human brain relevant to attentional and impulsive phenotypes. Hence, whilst *STS* could theoretically underlie sex differences in these domains, this idea remains to be formally tested.

There are multiple strands of evidence suggesting a role for steroid sulfatase in attentional and impulsivity phenotypes. First, it has recently been shown that *STS* is expressed in regions of the developing brain key to attentional and impulsive operations including the cerebral cortex, the thalamus and the basal ganglia i.e. regions whose structure/function is perturbed in ADHD (Stergiakouli et al., 2011). Second, males with deletions of the gene (or inactivating mutations within the gene) are at significantly increased risk of developing ADHD (notably the inattentive subtype) relative to the general population (Kent et al., 2008). Third, specific single nucleotide polymorphisms within the gene may be associated with an increased risk of developing ADHD, and an increased number of inattentive symptoms in ADHD cohorts (Brookes et al., 2008, 2010; Stergiakouli et al., 2011). Fourth, DHEA(S) levels are inversely correlated with ADHD symptomatology (Strous et al., 2001) and may be elevated by methylphenidate treatment (Maayan et al., 2003). Finally, mice with deletions encompassing the *STS* gene (or mice in which the enzyme's activity has been inhibited) show visuospatial attentional deficits, reduced levels of impulsive action (as indexed by performance on mouse variants of the 5-CSRT and SSRT tasks), elevated levels of aggression and increased locomotor activity (Davies et al., 2009; Nicolas et al., 2001; Trent et al., 2011; Humby et al., manuscript in preparation). Given this mounting experimental support, *STS* represents a strong candidate gene for neurocognitive deficits in TS, which have previously been mapped to Xp22.3 (Zinn et al., 2007).

To date, there is limited data on the neurobiological processes through which altered *STS* expression/function might mediate effects on attention and impulsivity. At the neurotransmitter level, rodent work has shown that steroid sulfatase inhibition may elicit elevated hippocampal acetylcholine release (Rhodes et al., 1997). Together with data showing that enzyme inhibition affects response accuracy in the visuospatial 5-CSRT task, we may speculate that steroid sulfatase activity is particularly important in orienting. Neurosteroids whose activity may be modulated by the steroid sulfatase enzyme could potentially modulate GABAergic and glutamatergic (NMDA) function (Dubrovsky, 2005; Zheng, 2009). Future work in model systems and humans should aim to examine how steroid sulfatase manipulations influence neurodevelopment, neurotransmitter balance and specific aspects of attention/impulsive behaviour.

### 6.3 MAOA

The X-linked *MAOA* gene (Xp11.3) encodes the mitochondrial enzyme monoamine oxidase A (MAOA) (Bach et al., 1988; Lan et al., 1989); this enzyme plays a vital role in the metabolism of monoamine neurotransmitters, catalysing the oxidisation of serotonin, noradrenaline and dopamine (Bortolato et al., 2008; Shih et al., 1999). The ability of monoamine oxidase to regulate monoaminergic systems makes it a prime candidate for effects on cognition and for vulnerability to disorders of attention and impulsivity such as ADHD. There is the possibility that MAOA expression/function is regulated in a sex-specific manner in one of two ways: (i) it may be a downstream effector for *SRY* in the brain, as described previously or (ii) it may partially escape X-inactivation in some tissues in humans; however, the data for this from studies using fibroblasts and hybrid cell work are inconclusive (Carrell and Willbard, 2005; Stabellini et al., 2009), and no comprehensive studies assaying escape from X-inactivation have yet been performed using discrete regions of the human brain.

As with steroid sulfatase, the evidence linking monoamine oxidase function with attentional, and particularly impulsive, phenotypes has come from a variety of sources: (i) inactivating mutations within the gene may lead to extreme impulsiveness and aggression in males (Brunner et al., 1993), whilst knockout of the *MAOA* gene in mice results in enhanced aggression (Cases et al., 1995), (ii) certain MAOA alleles may be associated with attention, impulsivity and aggression in healthy individuals, and may influence function of the anterior cingulate cortex (Fan et al., 2003b; Fossella et al., 2002; Manuck et al., 2000), (iii) association between *MAOA* polymorphisms (including the 30 base pair variable number tandem repeat sequence near the promoter) and vulnerability to ADHD (Gizer et al., 2009); these associations are typically heterogeneous across studies, and may be influenced by pervasive genotype–environment interactions (Kinnally et al., 2009) and (iv) the finding that methylphenidate, the predominant treatment for ADHD, inhibits monoamine oxidase activity (Solanto, 1998). There is some genetic evidence that the X-linked gene *MAOB* (Xp11.23) encoding the enzyme monoamine oxidase B (which is more selective than monoamine oxidase A and preferentially modulates degradation of dopamine), may also influence attention/impulsivity phenotypes and vulnerability to ADHD (Li et al., 2008), although the evidence that this gene may influence impulsivity is less strong than for *MAOA*. However, low levels of monoamine oxidase B activity in platelets have been associated with increased sensation seeking behaviours and impulsiveness (Oreland et al., 2002).

## 7 Sex-linked effects on autosomal genes

This review has focussed on sex-linked genetic mechanisms that may underlie attentional and impulsive phenotypes in mammals. However, there are without doubt a large number of autosomal genes whose products are likely to affect the phenotypes of interest here. It is possible that the expression/function of such autosomal genes may be modulated by sex, either by the products of sex-linked genes directly (e.g. *SRY* acting as a transcriptional activator for the autosomal *TH* gene), or by the downstream pathways influenced by sex-linked genes such as gonadal hormone systems. One interesting gene in this regard is *COMT* (22q11.21) which encodes the enzyme catechol-*O*-methyltransferase. *COMT* was implicated as a candidate gene for ADHD based on its high frontal cortex expression, its absence in ADHD-prone 22q11 deletion syndrome patients, and its role in catecholamine metabolism (Gizer et al., 2009). Most studies to date have failed to identify a significant association between *COMT* polymorphisms and ADHD; two studies have suggested that sex may moderate an association between the most intensively studied Val/Met polymorphism and ADHD, whereby possessing the methionine (Met) allele conferred risk of ADHD in boys, whereas possessing the valine (Val) allele conferred risk of developing ADHD in girls (Biederman et al., 2008; Qian et al., 2003). In further support, sex differences are often observed in the correlations between substance use, a neuropsychiatric disorder characterised by high levels of impulsivity, and the COMT Val108/158Met polymorphism (Tammimaki and Mannisto, 2010). The idea of an allele by gender interaction is consistent with observations that catechol-*O*-methyltransferase activity is greater in male prefrontal cortex (Chen et al., 2004) and that *COMT* homozygous knockout male mice have a twofold increase in dopamine levels in the prefrontal cortex compared with male wildtypes, whereas no such discrepancy occurs between female knockout mice and their wildtype counterparts (Gogos et al., 1998).

## 8 Summary and future work

There is persuasive evidence that the sexes differ with respect to their relative vulnerability to disorders of attention and impulsivity. Moreover, there is putative evidence that healthy males and females show differences in their attentional and impulsive profiles, and in the brain structures underlying these cognitive processes. However, these latter findings are, at present, inconsistent and the field requires further exploration. These sex differences in neurobiology must be underpinned by genes on the X and Y chromosomes exerting their effects by either direct action in the brain or via intermediary mechanisms such as systemic hormone secretion; here, we have reviewed several plausible mechanisms via which genes on the sex chromosomes may influence attention and impulsivity.

Future work, utilising state-of-the-art imaging techniques, should try to specify more accurately the neuropsychological and neuroanatomical differences that distinguish healthy males and females, male/female controls from their counterparts presenting with disorders of attention or impulsivity, controls from subjects with sex chromosome anomalies, and controls from subjects with mutations within specific sex-linked genes. To achieve the first two objectives, sex difference studies should ideally be planned *a priori* rather than analysing data from males and females *a posteriori*. Additional useful work in humans might focus upon identifying potential regions of interest underlying attention/impulsivity phenotypes on the X chromosome using large scale linkage or association or copy number variant analyses, although the idiosyncratic nature of this chromosome makes it difficult to study using standard methods (Ross et al., 2006; Schaffner, 2004). Finally, animal work may enable us to dissociate between sex-linked genes influencing attention/impulsivity directly, or via gonadal hormonal mechanisms (Arnold and Chen, 2009) and to examine the neurobiological systems underlying altered attention/impulsivity more intimately. Ultimately, identifying protective or risk factors encoded by sex-linked genes should enable us to develop more effective methods of treating sex-biased disorders of attention and impulsivity.
